# A Case Report of Spontaneous Unilateral Adrenal Hemorrhage in the Third Trimester of Pregnancy

**DOI:** 10.7759/cureus.93217

**Published:** 2025-09-25

**Authors:** Man Wai Cheng, Khurram Khan

**Affiliations:** 1 Internal Medicine, North Manchester General Hospital, Manchester University NHS Foundation Trust, Manchester, GBR

**Keywords:** abdominal pain in pregnancy, adrenal glands, adrenal insufficiency, left sided abdominal pain, unilateral adrenal haemorrhage

## Abstract

This case report describes a 33-year-old woman who developed spontaneous adrenal hemorrhage during the third trimester of pregnancy. She presented with left upper quadrant abdominal pain, which was described as pleuritic in nature, with shortness of breath. She also described nausea and had been sick a couple of times before she came to the hospital. However, she has remained hemodynamically stable. There were no signs of adrenal insufficiency. Adrenal hemorrhage was incidentally noted as she underwent investigation for suspected pulmonary embolism. Following the diagnosis of adrenal hemorrhage, she was managed conservatively under the joint care of physicians and the endocrinology team. A review of previous literature on adrenal hemorrhage in pregnancy was also included.

## Introduction

Different etiologies, like sepsis, trauma, coagulopathy, or previous surgery, could cause adrenal hemorrhage. However, pregnancy-related adrenal hemorrhage is a rare condition, reported in only 0.14% to 1.1% of pregnancies. Unilateral adrenal hemorrhage is more common than bilateral [1]. Patients may present with vague symptoms, and if no signs of adrenal insufficiency are present at the time of presentation, the diagnosis is often incidental while performing investigations and imaging to look for other causes of presentation, and often suspected as pulmonary embolism initially as was the case with our patient as well. Having a mortality risk of 15% in the general population, it is potentially life-threatening to both the mother and the fetus if not identified early [2]. This case report illustrates an idiopathic unilateral adrenal hemorrhage during the third trimester of pregnancy presenting with sudden onset, excruciating left upper quadrant and lower back pain, which was pleuritic in nature and was also associated with shortness of breath.

## Case presentation

A 33-year-old woman, 31 weeks into her fifth pregnancy, presented to the Emergency Department with a two-day history of sudden-onset left upper quadrant abdominal pain, associated with four to five episodes of vomiting on the same day. The pain was pleuritic with some shortness of breath. She did not describe any fever, cough, diarrhea, or urinary symptoms. She was hemodynamically stable. Physical examination showed tenderness over the left upper quadrant of the abdomen and left loin.  The chest was clear, the heart sounds were normal, and there was no lower limb swelling or tenderness.

In the initial investigations, white blood cell count was elevated, while C-reactive protein was normal. Hemoglobin level was slightly low. Liver and renal function tests were normal. Urinalysis showed positive WBC, and she was started on empirical cefalexin (Table 1).

**Table 1:** Biochemical investigations.

Biochemical investigations	Patient value	Reference range
White blood cell	17.6 x 10^9/L	4.0-11.0 x 10^9/L
Hemoglobin	108 g/L	120-150 g/L
Prothrombin time	0.5 second	9-13 seconds
Activated partial thromboplastin time	24.8 seconds	25-40 seconds
Lupus anticoagulation	Positive	Negative
Hepatitis screen	Negative	Negative
Urinalysis	White blood cell positive	Negative
Renin	2.8 nmol/L/hour	0.3-2.2 nmol/L/hour
Aldosterone	57 pmol/L	0-630 pmol/L

**Figure 1:**
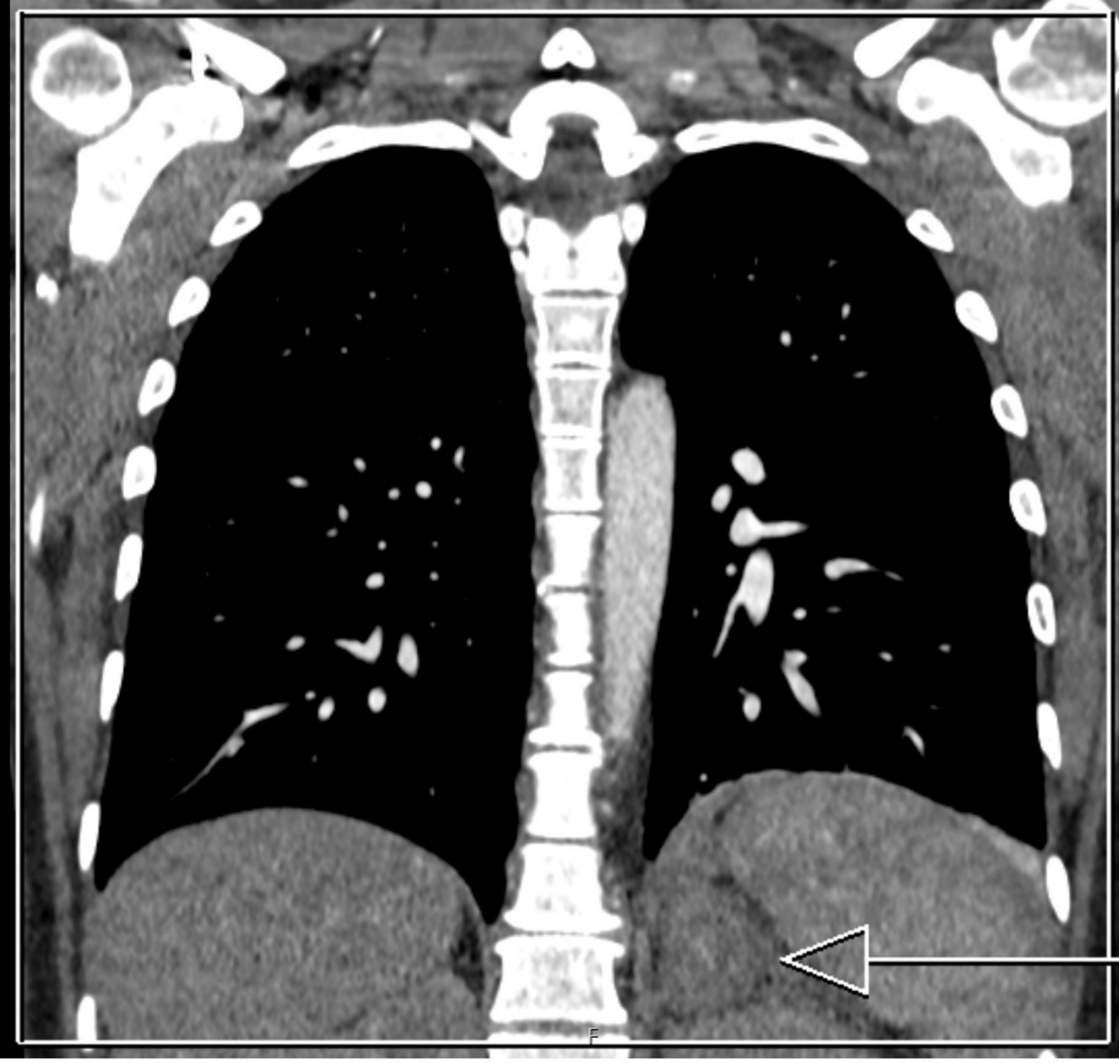
Coronal CT pulmonary angiogram showing an enlarged left adrenal gland (arrow).

**Figure 2:**
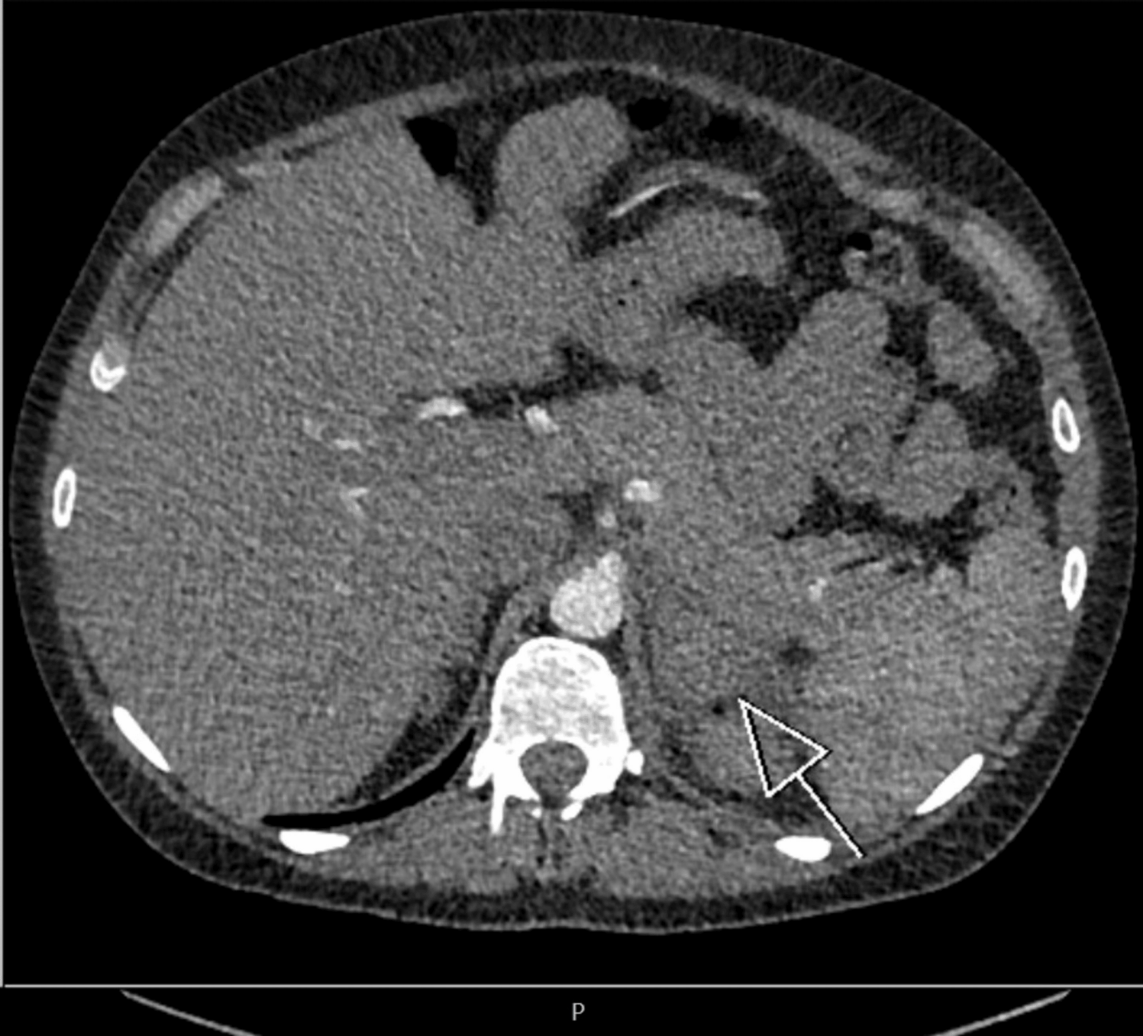
Axial CT pulmonary angiogram showing an enlarged left adrenal gland (arrow).

A CT pulmonary angiogram was arranged due to initial suspicion of pulmonary embolism. There was no pulmonary embolism, but the scan showed an asymmetrical left adrenal gland with increased size and density, radiologically concerning spontaneous adrenal hemorrhage involving the left adrenal gland (Figures 1-2).

**Figure 3:**
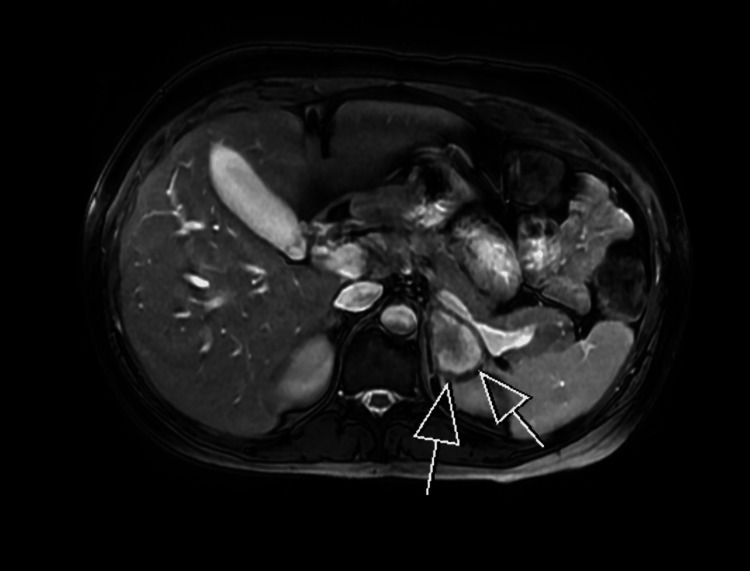
MRI adrenal showing an enlarged left adrenal gland.

Her vitals remained stable throughout, and hemoglobin levels were static. An adrenal MRI performed six days later showed an enlarged left adrenal gland, with no significant change in size compared to the recent CT, findings suspicious for adrenal hemorrhage and possible adrenal infarction (Figure 3).

Serial morning cortisol monitoring was performed and found to be adequate (Table 2).

**Table 2:** Cortisol level during admission.

Days after admission	Cortisol level (nmol/L)
Two	804
Three	584
Four	484
Seven	331
Eight	614

Cortisol levels had been adequate, and thus, she did not require a hydrocortisone supplement. She did not require any blood transfusion or surgical intervention due to a stable hemoglobin level. Her pain was managed by codeine. She was discharged from the hospital with a plan for a follow-up adrenal MRI and a short Synacthen test six to eight weeks postpartum. She eventually underwent a Cesarean section at 38 weeks due to breech presentation and experienced no complications. She did not attend the scheduled follow-up scan.

## Discussion

Managing abdominal pain in pregnancy can be challenging, first, because of the wide range of differential diagnoses, and second, due to not considering radiological investigation during pregnancy unless essential. In addition to obstetric causes, any etiology of abdominal pain of a normal adult must be considered. To complicate the process, certain conditions may be exacerbated by the physiological changes of pregnancy [3]. Adrenal hemorrhage is one of the important differentials. 

While adrenal hemorrhage occurring during pregnancy can be caused by different conditions like abortion, twisted ovarian cyst, antepartum hemorrhage, and coagulation disorders, these conditions often lead to bilateral hemorrhage [4]. In this case, a positive lupus anticoagulant was found. Aldaajani et al. described a case of antiphospholipid syndrome developing bilateral adrenal hemorrhage [5]. The patient had a history of a clot, but was already off anticoagulants at the time of presentation. The clotting time was not prolonged. She did not experience further clots and did not have recurrent miscarriages. The significance of this result is uncertain. The test should be repeated, and she should be referred to hematology or rheumatology for further investigations. The pathophysiology of unilateral spontaneous adrenal hemorrhage in pregnancy remains unclear; however, one hypothesis suggests that pregnancy-associated adrenal cortical hyperplasia and hypertrophy may lead to venous congestion, resulting in hemorrhage [6].

Symptoms can be non-specific, like the illustrated case, presenting only with abdominal pain due to capsular distension or sometimes due to infarction. On the other hand, patients may also present with more severe signs and symptoms like fever and shock, guarding, rigidity, or rebound tenderness of the abdomen [7]. One study showed that adrenal insufficiency was only present in 2% of the hemorrhage cases [8]. It may be because 15%-30% of adrenal tissue is possibly adequate to maintain adrenal function [9]. Nevertheless, the outcome of an undiagnosed adrenal hemorrhage is potentially fatal if there is ongoing bleeding and untreated adrenal insufficiency. Therefore, though the process toward diagnosis can be challenging, it is crucial to have this differential in mind. 

While MRI is the most sensitive and specific modality, it may not be readily available. An unenhanced CT without an adrenal washout phase can be used for the diagnosis of adrenal hemorrhage in acutely ill patients. MRI remains useful in surveillance for pregnant patients hoping to avoid radiation, as well as in cases where further characterization of the underlying mass is required. MRI is also helpful in determining the age of the hematoma. 

For bleeding within seven days, blood will be hyperintense on T1-weighted and hypointense on T2-weighted images. As time progresses, a hematoma more than seven weeks after presentation would be hypointense on both T1- and T2-weighted images [10]. 

Spontaneous adrenal hemorrhage can be treated conservatively in patients without adrenal insufficiency, as in the illustrated case, with pain management, monitoring of hemoglobin, and cortisol. It is essential to have close monitoring of the fetus. In patients who have unstable vitals or complications such as pre-eclampsia, preterm delivery may be indicated. In cases with ongoing bleeding, arterial embolization or even emergency adrenalectomy should be considered [11]. 

## Conclusions

Spontaneous adrenal hemorrhage in pregnancy can present with non-specific symptoms; therefore, the diagnosis is often not considered and may be missed until radiological investigations are performed to identify the cause of the pain. While incidence is low in pregnancy, it is an important differential to consider in patients with abdominal pain, as it can lead to mortality. CT adrenals are the suggested imaging modality in acute settings, while MRI adrenals can be used for follow-up and delineation of underlying masses if any are present. Stable patients can be managed conservatively by monitoring hemoglobin, cortisol level, and pain management. Patients with severe complications may need surgical intervention. This case report aims to highlight the importance of identifying adrenal hemorrhage in pregnancy in cases with a subtle presentation.
